# Organisational contextual aspects among medical social workers

**DOI:** 10.3389/fpsyg.2023.1196593

**Published:** 2023-07-13

**Authors:** Abdulaziz Albrithen, Nadir Yalli

**Affiliations:** ^1^United Arab Emirates University, Al-Ain, United Arab Emirates; ^2^Umm Al-Qura University, Makkah, Saudi Arabia

**Keywords:** social work, hospital social work, organisational structural, organisational resources, Saudi Arabia

## Abstract

Since the recognition of social work in health care, the number of social workers has dramatically increased. The proved association between psychosocial conditions and biophysical features encouraged in social work drove its professionalism and made social workers an essential part of the health care team in hospitals. Today, social workers are involved in medicine as specialised practitioners who share their knowledge and care along with other professionals to provide multi-disciplinary interventions and services to people in need. Using a self-administered questionnaires, this study aims to examine the relationship between two organisational-contextual aspects (i.e., organisational structural, and organisational resources) among social workers in health care in Saudi Arabia.

## Introduction

Social work practice in the medical filed forms part of patient care. Social work in medical settings is a growing field, and social workers continue to play a critical role in addressing the complex social and emotional needs of patients and their families.

Existing research indicates that hospital social workers perceive workplace limitations to practice concerning several aspects of their roles such as effectiveness (Michalski et al., 2000; Wong et al., 2000; Yip, 2004), productivity, efficiency and competence (Kayser et al., 2000; Mizrahi and Berger, 2001). The workplace issues open up an area of questioning related to organisational infrastructure.

Social work practitioners, as a professional group, are new entrants into the Saudi health care sector. They are largely employed in hospitals that are owned and operated by the Saudi Ministry of Health (SMoH), the main health care provider in the Kingdom. Hospital social workers are regarded by the Saudi government as important not only in supporting the health care organisation to effectively deal with illness-related psychosocial issues (Al-Saif, 1991), but also as vital human resources for implementing the national social and health development strategies.

Recent evidence from the Saudi literature suggests that, as in other countries, the Saudi health care sector is currently encountering challenges to keep abreast of changes in socio-economic conditions, with several studies highlighting barriers to health care professionals, particularly medical professionals, to achieving positive results and in maintaining efficiency (Al-Shammari and Khoja, 1992; Al-Rowais, 1996; Al-Aameri, 2000). Examples of reported difficulties include increased workload, inefficient inter-professional coordination and lack of time for teamwork, limited organisational resource for patients, cultural barriers and communication problems with patients, lack of professional development opportunities, stressful working conditions and ineffective administrative support (Al-Shammari and Khoja, 1992; Kalantan et al., 1999; Al-Ahmadi and Roland, 2005). The present research study, therefore, has conducted in order to provide literature though analysing the organisational aspects which influence their professional practice and personal conduct in the field.

This this study aims to explore practitioners’ perceptions in relation to the organisational factors that can influence their roles in the Saudi hospital sector, with special attention to issues such as current position structures and organisational commitment, resources available for social work.

## Significance of the study

Social workers are important members of the health care organisation and are an integral part in the delivery of efficient health care services (Saleh, 2002; Lymbery and Bulter, 2004). The position of social workers in Saudi health care is related to the fact that many professional practices in the Kingdom, including medicine, allied health professions and social work, have been largely adopted from Western industrialised countries (especially the United Kingdom and United States), and thus empirical findings from studies in these countries can be used to comprehend the overall function of hospital social workers and to demonstrate the workplace factors that may influence practitioners’ ability to effectively execute their duties. However, a review of existing international literature on health care issues and hospital social workers suggests that not all information is necessarily applicable or similar to the experience of Saudi practitioners. Saudi Arabia has its own unique socio-economic and political system and cultural climate, which may impact on many aspects of professional practice and shape the atmosphere in the health care system and characteristics of health care organisations. This in turn may produce workplace issues that could be similar to those reported by social workers in other countries or could be unique to Saudi practitioners. Given the crucial function in the delivery of health care, it is important, therefore, to understand the working context in which social work practitioners operate in Saudi hospitals, which may in turn highlight effective information that can enhance their effectiveness in health care interventions.

Social work practitioners can make an important contribution to their employing organisation and its overall ability to maintain effective and efficient performance. However, unless social workers are able to manage effectively the challenges and pressures that confront them within the organisation, they may be unable to fulfil their potential (Lymbery and Bulter, 2004).

An investigation of practitioners’ views, in relation to their roles and the issues that they perceive as influencing their functions in Saudi hospitals, can produce valuable insights which could contribute to the development of effective strategies to enhance social workers’ ability to implement workplace plans and support their organisations to achieve objectives. This is the salient situation in Saudi Arabia, where professionals and organisations, including in the public health care sector, are expected to work actively and efficiently to strengthen people’s and communities’ capacities to respond to the nation’s development objectives and to overcome undesirable circumstances that may accompany the rapid transitions in the Kingdom’s social and health conditions (Al-Qurni, 2003).

It was the intention of this research, therefore, to delineate the experience of social work practitioners within Saudi hospitals by exploring organisational contextual aspects among social workers. This is an important focus of this study to identifying areas for future improvement.

## Methodology

A self-administered questionnaire was designed using a Likert scale and free response questions organised in three sections (i.e., demographical data, organisational factors, and job frustration). The research tool was dispensed to 219 Saudi social workers who practice at state hospitals.

The instrument’s reliability of the questionnaire was measured using multiple choice tests of Cronbach’s Alpha. The studies involving human participants were reviewed and approved by the Research Ethics Committee of the Department of Social Work at UQU, prior to starting the data collection procedures. Also, written informed consent to participate in this study was provided by the participants. The Statistical Package of Social Sciences (SPSS) software, version 26 for Windows, was used to analyse the date as well as calculate the reliability of the instrument. All sections concerning organisational structure and resources had a Cronbach’s Alpha ranging from 0.86 to 0.94 (i.e., acceptable reliability).

This study concerns the quantitative analysis regarding organisational factors which influence the performance of social work roles in Saudi hospitals. For each question participants were asked to indicate their agreement or disagreement, on a five point Likert-Scale ranging from (1) strongly disagree to (5) strongly agree, with positive and negative statements related to organisational issues. The study explores responses related to organisational-contextual issues in the hospital, such as organisational structure and resources. Descriptive analysis was carried out to explore the answers; therefore and for analytical purposes, responses to the negative statements in each part of the questionnaire were accounted so that participants who scored low (1–2) show unfavourable perceptions of their organisations, while those scored higher on the re-coded scale (i.e., 4–5) indicated to report positive answers. The reverse coded items are designated with the letter [R] in each table. The findings are organised and summarised in frequency tables which illustrate category distribution for participants’ answers.

## Demographical data

The demographic findings show that over half of the participants were male (*N* = 131, 59.8%), and 87 (39.7%) were female. The age of the respondents ranged from 24 to over 51 years old, with a mean of 36. Practitioner’s years of experience working as hospital social workers ranged from less than 1 year (0.2 months) to 15 and more years, with a mean of 10.64 years and a median of 10.00 years. The majority of respondents (*N* = 139, 64.5%) held bachelor degrees, 30 (13.5%) held bachelor degrees with a diploma, 26 (11.2%) held master’s degrees, and 24 (10.8%) described their educational level as ‘other’. Almost two thirds of the participants (*N* = 144, 65.8%) were in a permanent post, while 75 (34.2%) were on short-term contracts (Table 1).

**Table 1:** Responses demographical data (*N* = 219).

		*N*	%
Gender	Male	131	59.8
	Female	87	39.7
Age	24–30	32	14.6
	31–40	65	29.6
	41–50	96	43.9
	51 and +	26	11.9
Years of Experience	Less 1 year	11	5.0
	2–5 years	44	20.0
	6–10 years	66	30.2
	10–15 years	65	29.8
	15 and more	33	15.0
Qualification	Bachelor	139	64.5
	Bachelor + Diploma	30	13.5
	Master	26	11.2
	Other	24	10.8
Employment type	Permanent employ	144	65.8
	Short-term	75	34.2

## Findings

Participants’ responses in relation to the current organisational aspects of their professional practice in the Saudi hospital sector are analysed and described, which is divided into two sections relating to organisational structural issues, and organisational resources.

### Perceived organisational structural issues

This segment examines the responses of participants in accordance to eleven statements pertaining to structural aspects of the workplace which provides the background for professional practice in the hospital. As elucidated by the results summarized in Table 2, social workers often report difficulties in completing their tasks due to perceived lack of authority possessed by social workers in the broader context of healthcare. Bolstered by the findings present are previous international publications (Cherniss, 1980; Arches, 1991; Balloch et al., 1998; Reid et al., 1999; Wong et al., 2000) and expand upon the understanding of the ramifications of interrelated structural characteristics on social work practice within the context of Saudi Arabia.

**Table 2:** Responses and results related to organisational-structural issues.

	Mean	S.D.	Negative score (1–2)		Neutral score (3)		Positive score (4–5)	
			*N*	%	*N*	%	*N*	%
1. Mostly, I have little to do in this setting	2.33	1.14	143	65.3	39	17.8	36	16.4
2. I have similar authority to medical professionals	2.34	1.33	145	66.2	20	9.1	53	24.2
3. Mostly, I have little to advocate for the rights of my clients [R]	2.35	1.19	137	62.6	35	16.0	45	20.5
4. I can participate in the internal governance of the organisation	2.42	1.30	137	62.5	29	13.2	51	23.2
5. Mostly, I receive encouragements to come up better ways of handling things	2.49	1.30	134	61.2	31	14.2	54	24.7
6. I feel that the rules prevent me from satisfying job requirements [R]	2.53	1.13	126	57.5	38	17.4	55	25.2
7. Policies are good for effective response to social issues	2.54	1.17	126	57.5	37	16.9	54	24.6
8. I easily utilise the hospital services to do my professional job	2.64	1.28	124	56.6	30	13.7	63	28.8
9. Mostly, I have to refer all matters to someone higher up [R]	2.72	1.21	112	51.2	43	19.6	63	18.7
10. I view the guidelines of SW in the hospital are not clear [R]	2.81	1.23	100	45.7	45	20.5	71	32.4
11. The hospital treats SWs as professionals important to patient care	2.83	1.33	107	48.9	32	14.6	80	36.5
12. I have clearly defined goals, tasks and responsibilities	2.96	1.34	92	42.0	35	16.0	92	42.0
13. Mostly, all my works current concerned with routine matters [R]	2.97	1.21	93	42.5	46	21.0	80	36.5
14. Mostly, all my works are following orders [R]	3.00	1.28	87	39.8	39	17.8	90	41.1
15. SW department is strongly connected to other departments and units in the hospital	3.35	1.12	55	25.1	56	25.6	108	49.3

[R] Recoded statements.

In this research, role structure alludes to the way in which the present duties of social workers are conceived, allocated, and identified within the health-care organization. Illustrated by the data presented in Table 2, participants were inclined to view their roles as dubious and ill-suited for successful practice.

65.3% (*N* = 143) of respondents (nearly two thirds) disagreed with the statement “Mostly, I have little to do in this setting.” In contrast only 16.45 (36 participants) agreed. A consequence of this could be the administrative among other responsibilities assigned to social workers that have little to do with social work in Saudi hospitals. Also suggested by the data is that the practitioners are allocated more duties which are outside of their professional domain (Al-Saif, 1991). This may indicate that the social work profession in Saudi Arabia is still in its’ advent. Current job guidelines, issued by hospitals, impart to the view among practitioners that their workplace stifle effective application (e.g., lack of coherent job descriptions) as supported by participants’ answers.

The second table (Table 2) reveals that an overwhelming number of participants (*N* = 137, 62.7%) in this report assert that social workers lack power to be true proponents of the rights of their clients and to meet the practice requirements. In addition, 92 respondents (42%) voiced that they have clearly established job responsibilities and objectives to achieve while the same number of participants (*N* = 92, 42%) believe otherwise. Only 16% of participants (35 in total) answered impartial. A way to explain this discrepancy may be the differing educational background and previous job experiences among the surveyed social work staff. According to nearly half of the respondents (*N* = 100, 45.7%), current job guidelines for social work are indefinite while 32.4% (71 participants) asserted the opposite. 20.5% of the (45) participants were impartial according to Table 2. Further examination using the cross-tabulation method display that 66 respondents who believed their job guidelines were ambiguous also stated their responsibility and social work objectives were not clearly delineated. This is compared to 46 of total participants who expressed that they have transparent job instructions and responsibilities. This draws attention to the issue of vagueness in the workplace (e.g., lack of clear job outlines and expectations) which has been recognized in international research as an unexceptional systematic obstacle for social work practitioner in the hospital sector (Balloch et al., 1998; Reid et al., 1999; Wong et al., 2000).

On an international scale, the role of vagueness in social work practice has been linked to social workers’ beliefs that their department are in need of effective connections with other sections of the hospital that both directly and indirectly influence their practice (Cherniss, 1980; Arches, 1991). 25.1% (*N* = 55) of participants did not agree with the item “SW department is strongly connected to other departments and units in the hospital” while nearly 50 % of participants (*N* = 108, 49.3%) agreed. Further analysis utilizing cross-tabulation for responses regarding other groups and personnel whom they are in communication within order to complete responsibilities suggests a limitation in social work relations with other vital units in the organization, especially the clinic management division. An attainable argument is that participants’ answers relating to their department associations to other units may be based on the social workers’ perspective in relation to their own attempts to instigate and uphold these connections with different units and their employees in the hospital. Nevertheless, this may not automatically mean that these relationships are particularly firm or beneficial for the social work department within the workplace.

This circumstance could be further illuminated by their judgment of hospital attitudes towards the significance of their professional responsibilities within the organization. Plenty of participants (*N* = 107, 48.7%) disputed with the assertation that their hospital views social workers as important to provide professional care. This viewpoint evokes other literature, as (Huxley et al., 2005: 1076) examine that social workers may compare “themselves with health service colleagues, who are privileged in many respects,” chiefly in connection to working arrangements and conditions. Therefore, it is possible to contend that many social workers reckon that their labor is undervalued and disregarded by their employers and other staff in the workplace. This is connected to insight of an obscure organizational position in the hospital setting (Reid et al., 1999; Wong et al., 2000). However, more than one third (*N* = 80, 36.5%) of participants were in accord with the above statement suggesting that some social workers feel appreciated and respected by the organization. From a cross-tabulation analysis of participants’ responses concerning the extent to which they were treated as important professionals in health care and their answers in relation to clarity of their job responsibilities in the hospital, respondents who reported that the hospital regards them as being important in relation to patient care are likely to the be the one who perceive job clarity (*N* = 52), while those who felt devaluated by the employers are likely to be those respondents with high perceptions of role ambiguity (*N* = 69).

As organisations, hospitals have their own policies which regulate personnel behaviour and control service procedures. According to Table 2, organisational rules in the hospital appear to be a problematic issue for social workers. Ninety one participants (41.1%) indicated that what they are mostly doing in their workplace is following orders from higher authorities in the hospital, while about 87 (39.8%) felt otherwise. This finding suggests that some hospital social workers do not feel complete autonomy to operate in the manner which their professional expertise might prescribe (Al-Saif, 1991), which impacted on their perceptions that their job was routinised (Lymbery and Bulter, 2004). Ninety three (42.5%) of the participants described their job as mainly concerned with routine matters, while 80 (36.5%) did not believe so. Findings also show that social workers encounter barriers to the effective performance of their job due to the highly formalised and inflexible policies of the hospital, as evidenced by the responses of many participants in this study. More than half of participants (*N* = 112, 51.2%) concurred that “I have to refer all matters to someone higher up” within the organization. Only 18.7% (*N* = 63) dissented, possibly reflecting the bureaucratic direction of Saudi hospitals (Al-Saif, 1991). This often involves obdurate rules possessing the ability to restrict professionals’ ability to oversee their labor (Arches, 1991; Crozier, 2017). Again, over half of the respondents (*N* = 126, 57.5%) relate that workplace rules are inept and unsuitable to the facilitation of improved social services. Only 24.6% (54 participants) affirmed that “Policies are good for effective response to social issues.” Moreover, rules were also viewed as contradictory to social work requirements, with most participants (*N* = 126, 57.5%) referring to the rules structure within their organisation as a preventing factor in satisfying job requirements, while one quarter (*N* = 55, 25.2%) did not believe this to be so. A cross-tabulation analysis of participants’ responses in relation to the effectiveness of work-related policies and their opinions of organisational rules as inhibiting effective practice indicates that most (*N* = 96, 76.2%) of those who believe that the design of services in the organisation is inefficient also state that current organisational rules are problematic to satisfying job needs.

Saudi hospitals are structured from the top-down with power and authority centralized (Al-Saif, 1991). The assumption is that this extreme bureaucracy is hindering social workers’ involvement in the organizing to its’ own detriment as greater than half of participants (*N* = 137, 62.5%) reported difficulty in internal governance participation. In contrast, only 23.2% (51 participants in total) claimed they can participate. According to Table 2, social workers possess little to no control over their roles as compared to other health-professionals in the hospital, they lack authorization to do their job. This in turn highlights the unequal distribution of authority and power among the organisation’s units, especially with the dominance of medical practitioners. Nearly two thirds of the participants (*N* = 145, 66.2%) stated that they do not have similar authority to that of medical professionals, while slightly less than a quarter (*N* = 53, 24.2%) believed the contrary. The lack of necessary power and authority given to social work staff seems to deter them from assuming innovative practice and trying new things. Most of respondents (*N* = 134, 61.2%) expressed disagreement with the statement worded “I received encouragements to come up with better ways of handling things,” and approximately only one quarter (*N* = 54, 24.7%) agreed with this statement. This implies that participants encounter adversity in utilizing their professional skills and techniques to assist their clients.

### Perceived issues relating to organisational resources

This segment discusses social workers’ insight in relation to the resources accessible for aiding them in performing their duties. In Table 3 below, there is a summarization of participants responses to ten statements related to resources in the workplace. Want of funding and organizational resources have been cited in global literature as one of the major troublesome aspects for social workers in the hospital sector (Michalski et al., 1999; Kayser et al., 1999; Kayser et al., 2000; Rushton, 2000; Huxley et al., 2005). Comparably, Saudi social workers seem to understand the restrictions in their roles consequently to limitations in resources in state hospitals (Al-Shammari and Khoja, 1992). As elucidated by Table 3, hospital workers perceive that there is underfunding for their services. This is corroborated with the majority of participants (*N* = 183, 83.6%) reporting that their department are underfunded. Possibly peculiar to social workers in Saudi hospitals is that they are also denied the freedom to appropriately cope with financial hurdles through discovering alternative resource funding (Arches, 1991).

**Table 3:** Responses and results related to organisational resources issues.

	Mean	S.D.	Negative Score (1–2)		Neutral score (3)		Positive score (4–5)	
			*N*	%	*N*	%	*N*	%
1. There should be a small department of SW within all patients’ units [R]	1.47	0.860	200	91.3	8	3.7	10	4.6
2. Our SW department is receiving good funds	1.72	0.954	183	83.6	23	10.5	13	6.0
3. SWs have enough flexibility to manage the financial affairs of their department	1.74	0.905	182	83.1	26	11.9	11	1.5
4. A shift working hours’ system could solve the shortage of SWs [R]	2.27	1.183	149	68	31	14.2	39	17.8
5. SWs have more of input into planning related to funding and resources	2.31	1.220	144	65.8	23	10.5	51	23.3
6. We assess patients’ needs but we mostly we feel that we have to tell them about our limited resources [R]	2.37	1.074	139	63.0	44	20.1	38	16.9
7. We have our own allocated space	2.39	1.330	133	60.7	29	13.2	56	25.6
8. The hospital requires support services that are unavailable [R]	2.41	1.123	132	60.3	47	21.5	40	18.2
9. There is less space in which to carry out professional social services [R]	2.67	1.355	130	59.4	23	10.5	66	30.1
10. There are inadequate staffing levels within SW department [R]	2.80	1.338	109	49.7	27	12.3	83	37.9

[R] = Negative recorded statements.

Upwards of 80% respondents (*N* = 182) demonstrated that they lack the autonomy to manage the monetary concerns of their department. Highlighted by further analysis is the role of governing policies of the hospital which look to reinforce the lack of freedom for workers of the social work department. Illustrative of this is that more than two-thirds (*N* = 124, 68.1%) of participants who argued that they have bounded freedom to manage the financial matters of their department in addition felt that the encouragement to suggest improvement in workplace practices were limited as well.

Further indicated by Table 3 is that participants believe they have restricted participation in the planning and decision-making process related to allocating resources to and funding of social work departments. The preponderance of respondents (*N* = 144, 64.8%) were at odds with the statement “Social workers have more of input into planning related to funding and resources,” while a mere 23.3% (51 participations) were in correlation. The centralization in Saudi health-care sector may attribute to this as it is associated with the general assumption within the hospital sector that all decisions relevant to the organization’s resources and tasks are the principal functions of the management system (Al-Saif, 1991). In general, the extreme centralization of both arbitrary and vital decision-making is considered inimical to patients’ care and the overall operations of the health-care sector. The extent to which social workers believe their thoughts and perspectives are considered in the larger planning and decision-making process pertinent to resources were cross tabulated with their answers regarding their capacity for involvement in internal governance of the organization in these findings. This exposes that most participants (*N* = 98, 68.5%) who communicated that they have narrow input into organizing and decision-making connected to resources furthermore consider themselves barred from participation in internal governance of the organization, unable to negotiate their needs as workers and contribute to decisions directly impacting their services (Daft, 2015).

In order to efficiently complete the assortment of group and community services necessary for successful intervention and alleviation of health and social problems, the availability of space to social workers cannot be omitted. To illustrate, higher availability of suitable program amenities such as group therapy, self-help circles, and individual support can properly assist patients such as those with life-threatening diseases, chronic illnesses, or addictive disorders alongside their caregivers to develop coping skills and means of improving current conditions (Atkinson, 1997; Alston and McKinnon, 2001; Payne, 2006). Many respondents contended that the availability of space and support facilities in which to perform their tasks were lacking. Over half of the participants (*N* = 130, 59.4%) reported that in their department there is a very restricted amount of space to carry out compulsory social services such as community practice according to Table 3. However, 30% of participants (sixty-six in total), presume otherwise. Inconsistencies in space allocation to social work ministries across the overall hospital sector is suggested by this finding.

Also expressed by the results were the lack of private spaces and necessary supplies such as desks, computers, telephones, and other office equipment for social workers within the organization. The statement “We have our own allocated space in the hospital” prompted much disagreement among the respondents (*N* = 133, 60.7%) while scarcely over one quarter (*N* = 56, 25.6%) were in accordance with that assertation. It is possible that there has been an oversight to the differences between the needs of social workers and medical staff/professionals (including nurses and physiotherapists), one being the essentiality of space in order to successfully complete assigned tasks. It is crucial to document that most social work proceedings such as interviewing and assessing clienteles, collaborating and organizing with groups within the community, require an area to execute these activities. By contrast, many duties done by the healthcare professionals can be completed without specified spaces. In addition, a prerequisite to productive social work is confidentiality between patient and social worker which necessitates privacy; without privacy, it will be difficult in gaining a patient’s trust (Al-Shammari and Khoja, 1992). Sanctioning privacy within social work is obliged to extend into administrative matters such as patients’ notes and record-keeping. This alone demonstrates the need for private spaces as absence of private spaces alongside other necessary supplies factors in participants’ negative sentiments surrounding the workplace. That the workplace does not regard them as vital professionals in the administration of health care in their communities. There is a clear correlation between lack of private spaces and feelings of disregard by employers cited in the study as a substantial proportion (*N* = 80) of respondents who reported lack of private spaces also expressed that they felt their directors ignore their importance to patients’ welfare.

The respondents consider their profession as important to all patient-care units and, therefore, they believe that the hospital should offer them a space in all internal wards and out-patient units. The majority of participants (*N* = 200, 91.3%) indicated that “There should be a small department of SW within all patients’ units of the hospital,” while only 10 (4.6%) reported disagreement, (mean score 1.47, SD 0.860). Given the extremely high proportion of respondents who were in agreement with the above statement, it can be concluded that social workers believe that they lack both enough space and suitable areas to perform their roles in a satisfactory manner.

Staffing levels within the social work department were also viewed by many respondents as poor. Nearly half of the participants (*N* = 109, 49.7%) agreed that inadequate staffing levels within their departments resulted in confining them to simple tasks, while 83 (37.9%) did not agree. This relatively high proportion who were positive regarding staffing levels suggests that there may be inconsistencies of staffing allocation across the sector. In this regard, it is important to mention that there were approximately 280 social workers operating in more than 37 hospitals in western Saudi Arabia in 2018, most of which were of a large size with 300 or more beds per hospital (Saudi Ministry of Health [SMoH], 2019). This means that a social worker is assigned to work with a minimum of 30 beds (or patients), in addition to his/her other duties in the hospital.

Accordingly, participants’ perceptions relating to staffing levels were cross-tabulated with their answers concerning the amount of their workload, to examine the relationship between the two variables. A considerable number of respondents (*N* = 71) perceive difficulties in managing an organised job in the hospital due to inadequate staffing and high workload. This finding also confirm results from previous sections in that professionals are more involved in the provision of administrative tasks (e.g., clerical work, etc) or other non-social work duties.

Furthermore, these discoveries accentuate the correlation between the adjudged labor shortage, workload, and the shift system within the community service ministry. As evinced by Table 3, professionals deem the shift-work system as governing factor in staff capacity. Over two thirds of respondents (*N* = 149, 68%) asserted that working hours in reciprocation to the paucity of social workers should not exceed two shift periods while in contrast a mere 17.8% (39) of the total number of respondents maintained otherwise. A three-shift system per day, with approximately 8 h duration per shift seems to be the standard work structure despite the variation of working hours within a hospital service ministry due to individual hospital stipulations. Social workers may rotate periodically between three shifts or work one shift exclusively. Difficulties of implementing the shift-system are evident given the reality of the general scarcity of hospital social workers in Saudi Arabia with the consequence being the restriction of hospitals to a meager eleven practitioners staffing in their social work department. Worsening already dire circumstances is the strict gender segregation whereby social workers are confined to dealing with patients of their own gender. This consequently leads to severe shortage of work for some while other individuals are inundated with caseloads. The study’s results exhibit that the majority of respondents on individual working schedules agree that in order to avoid staffing conflicts department workhours should not exceed two working periods. Over 42% of those in agreement with this sentiment were working on a day shift. Feasibly, this is due to the long, arduous workloads during peak hours which correlates with the contention that if working periods were curtailed it would greatly improve worker performance.

#### Organisational structure and organisational commitment

Organisational structural aspects in the workplace have been linked to social work practitioners’ attitudes and their overall commitment to the employing organisation. Commitment to the workplace generally refers to the “employees’ emotional attachment to, identification with, and involvement in, the organization” (Meyer et al., 1998: 32). Many international studies illustrate that organisational structure and resources can impact on practitioners’ commitment to their employers (Mowday et al., 1979; Rabin and Zelner, 1992; Abu-Bader, 2000; Allen et al., 2004). For example, in a study conducted by Rabin and Zelner (1992), the researchers reported that a lack of job clarity predicts a high turnover.

The findings of this study highlight a statistical association between participants’ views concerning the organisational structural conditions in which they operate and their responses in relation to organisational commitment. As illustrated in Table 4, participants’ responses concerning organisational commitment (i.e., ‘I am proud to work for this organisation’) were correlated with their views concerning the extent to which their job responsibilities and goals are clearly defined (*r* = 0.42, *p* ≤ 0.01) and the extent to which they feel existing job guidelines are vague (*r* = −0.33, *p* ≤ 0.01). These correlations indicate that participants who perceived clarity on the job were more likely to report a positive relationship with their employing organisation. One reason for this is related to the quality of formal supervision in the social work department (Mizrahi and Abramson, 1985; Wong et al., 2000; Yip, 2004). For example, newly employed practitioners, who work within a social work department that is headed by highly-qualified, specialised and experienced supervisors, may find it less difficult to alleviate uncertainty and understand their workplace requirements without the need to rely on written documents, such as job descriptions and guidelines, which in turn can foster a more positive attitude towards the organisation than their peers in other hospitals who may not have access to an experienced/professional supervisor.

**Table 4:** Correlation results concerning organisational structure, resources, and organisational commitment.

Questionnaire item	Organisational commitment
Organisational structure	
*Job clarity*	
I have clearly defined goals, responsibilities and tasks	0.42 (**)
I view the guidelines of SW in the hospital are not clear	−0.33 (**)
*Job autonomy*	
Mostly, all my works current concerned with routine matters	−0.38 (**)
Mostly, all my works are following orders	−0.42 (**)
I have to refer all matters to someone higher up	−0.31 (**)
*Professional linkages*	
The hospital treats social workers as professionals important to patient care	0.42 (**)
My department is strongly connected to other departments in the hospital	0.35 (**)
Organisational resources	
*Workload*	
There are inadequate staffing levels within SW department [R]	−0.28 (**)

^*^Correlation is significant at the 0.05 level (2-tailed). ^**^Correlation is significant at the 0.01 level (2-tailed).

The statistical data of this study also indicate a relationship between respondents’ self-reported organisational commitment and their perceptions of job autonomy in the workplace (Allen et al., 2004). Table 4 shows significant associations between participants’ responses concerning commitment to their place of employment and their answers to the statements ‘Mostly, all my works are following orders from persons in higher positions in the organisation’ (*r* = −0.42, *p* ≤ 0.01), ‘Mostly, all my works current concerned with routine matters’ (*r* = −0.38, *p* ≤ 0.01), and ‘All matters have to be referred to someone higher up for a final answer and approval’ (*r* = −0.31, *p* ≤ 0.01). These correlations indicate that the extent to which participants perceive autonomy in their practice has a significant impact on their commitment to their workplace. In other words, participants who perceived little autonomy to do their job were more likely to report less commitment to the organisation.

According to the findings, participants’ self-reported organisational commitment was also correlated to their perceptions of their professional linkages in the health care organisation. Table 4 illustrates a significant statistical association between participants’ reported commitment to the organisation and the extent to which they believe that they are treated as professionals important to patient care (*r* = 0.42, *p* ≤ 0.01) and the extent to which they think that their department is well connected to other departments in the work environment (*r* = 0.35, *p* ≤ 0.01). These positive correlations indicate that participants who viewed their professional linkages as effective were more likely to report a strong commitment to their employing hospitals.

#### Organisational resources and organisational commitment

The statistical analysis of the data from this study also highlights a significant association between participants’ responses concerning organisational commitment and their perceptions of the impact of the hospital resource aspects on their workload. Table 4 shows that self-reported commitment to the workplace was correlated with responses to the statement ‘There are inadequate staffing levels within SW department confine SWs to simple tasks’ (*r* = −0.28, *p* ≤ 0.01). This negative correlation indicates that participants who viewed their workload in a negative light were more likely to indicate a low commitment to their employing organisation. The association between workload and organisational commitment can be linked to variation among participants with respect to employment features and benefits such as salary. The findings of this study, for instance, show a significant association (*r* = 0.19, *p* ≤ 0.05) between salary, organisational resources and organisational commitment. Accordingly, it is possible that participants with low salaries may find it more difficult to accept additional work than those with higher salaries, which can impact on their relationship with their workplace (Abu-Bader, 2000). It is also possible that younger practitioners may perceive more difficulties in managing workload which, in turn, may affect their perceptions of ineffective commitment to their employing organisation.

### Regression analysis: organisational determinants of organisational commitment

The findings from the correlation results were used to inform the further examination of the data using regression analysis to delineate the most important organisational aspects that contribute to practitioners’ personal experiences in the hospital environment. For regression analysis purposes, structural and organisational resource items were organised into four independent/predictor categories that were labelled: *job clarity* (‘I have clearly defined goals, responsibilities and tasks’, and ‘I view the guidelines of SW in the hospital are not clear’); *job autonomy* (‘Mostly, all my works current concerned with routine matters’, ‘Mostly, all my works are following orders’, and ‘All matters have to be referred to someone higher up’); *professional linkages* (‘The hospital treats social workers as professionals important to patient care’, and ‘SW department is strongly connected to other departments and units in the hospital’); and *workload* (‘There are inadequate staffing levels within SW department confine SWs to simple tasks’). This is because some of these questionnaire items were similar in meaning and/or had been originally designed to measure certain aspects concerning organisational structure (e.g., role structure) and resources (e.g., staffing) in the workplace. Additionally, this procedure had enabled the computation of the mean scores for variable groups, with two items and more, that were subsequently used for the regression analysis with the dependent variable (organisational commitment), while controlling the individual-demographic variables (personal/professional characteristics).

Accordingly, a regression analysis was undertaken to estimate and develop model predicting organisational commitment in social workers. The order in which the variables were entered into the model is justified by the key factors identified in previous literature and by the findings of this study. In the analysis, the following variables were entered as the independent variables: Job autonomy, job clarity, professional linkage, workload, location, age, major, years of experience, and salary. The dependent variable was organisational commitment.

#### Predictors of organisational commitment

The statistical data presented in Table 5 below illustrates that 14% of the variance in organisational commitment is explained by variance in the set of model predictors. According to Table 5, two factors were highlighted as significant predictors in organisational commitment: job autonomy and job clarity. The results identified job autonomy as the most important contributing factor to organisational commitment (*B* = 0.346, *t* = 3.445, *p* = 0.001). This finding indicates that as job autonomy increases, organisational commitment increases. This finding may not be consistent with previous work in the international literature (Abu-Bader, 2000; Allen et al., 2004). In their study, Allen et al., for example, reported that job autonomy did not have a significant effect on organisational commitment. The findings of this study may relate to the specific context in which inexperienced practitioners operate in a highly centralised medical system in Saudi Arabia, which impacts on their perceptions of low autonomy in the workplace and, accordingly, on their opinions of ineffective relationships with their employing organisations.

**Table 5:** Regression results for structural and resource organisational aspects: predictor of organisational commitment.

Model	*R*	*R* square	Adjusted *R* square	Std. error of the estimate	*F*	*P*
2	0.374	0.140	0.131	1.193	15.163	<0.000

	Unstandardised coefficients	Standardised coefficients			
Model	B	Std. error	Beta	*t*	Sig.
(Constant)	1.454	0.302		4.814	0.000
Job autonomy	0.296	0.098	0.227	3.034	0.003
Job clarity	0.255	0.088	0.218	2.908	0.004

According to the findings of this study, job clarity was also identified as the second most important predictor (*B* = 0.209, *t* = 2.338, *p* = 0.020) of organisational commitment. This finding indicates that as clarity on the job increases, organisational commitment is more likely to increase (Joseph and Conrad, 1989). In this study, neither professional linkages nor workload had a significant effect on organisational commitment. Additionally, none of the respondents’ individual characteristics were identified as important predictors of organisational commitment in this study.

## Conclusion

The organisational-contextual was related to organisational circumstances that were perceived to impact on the scope, content and directions of practitioners’ everyday practice in the hospital and incorporate the following:

1. Organisational structural obstacles, including:
  - Role/job design problems (e.g., lack of clear job guidelines and increased involvement with less professional work).
  - Constraints related to rule/work-regulations structure in the workplace (e.g., inflexible work-related politics, rigid bureaucracy), which were perceived to impact on practitioners’ professional autonomy on the job and to decrease the amount of their client work.
  - Barriers concerning power structure in the hospital such as outmoded hierarchy and centralisation of authority, which were perceived to impact on practitioners’ linkages with other units in the workplace and to limit their ability to participate in decisions related to their services and the work of the organisation.
2. Organisational resources constraints were related to the lack of funding for services, inadequate staffing levels in the social work department, insufficient space and necessary work-related equipment and means, and lack of support services in the workplace for psychosocial care; all of which were perceived by respondents to create difficulties for social work staff to practise their roles in an efficient manner (e.g., increased workload).

The findings from the analysis concerning the relationship between respondents’ perceptions of organisational structure and resource issues in the workplace and participants’ self-reported reactions toward the job. Using correlation analysis, the results highlight significant statistical associations between participants’ opinions concerning organisational structure and resources that are related to job clarity, job autonomy, professional linkages and workload, and respondents’ feelings of their self-reported commitment to the employing organisation.

Using the above-mentioned set of identified variables (i.e., job clarity, job autonomy, professional linkages and workload), a regression analysis was performed to identify the most important predictor of organisational commitment. According to the findings, organisational commitment can be determined by the extent to which they perceive adequate autonomy to carry out their roles and the extent to which they perceive clarity in the job in general. In other words, lack of clarity on the job and decreased job autonomy are responsible factor for decreased organisational commitment among participants in this study.

## Data availability statement

The raw data supporting the conclusions of this article will be made available by the authors, without undue reservation.

## Ethics statement

The studies involving human participants were reviewed and approved by the Research Ethics Committee of the Department of Social Work at UQU, prior to starting the data collection procedures. Also, written informed consent to participate in this study was provided by the participants.

## Author contributions

All authors listed have made a substantial, direct, and intellectual contribution to the work, and approved it for publication.

## Conflict of interest

The authors declare that they have no known competing financial interests or personal relationships that could have appeared to influence the work reported in this paper.

## Publisher’s note

All claims expressed in this article are solely those of the authors and do not necessarily represent those of their affiliated organizations, or those of the publisher, the editors and the reviewers. Any product that may be evaluated in this article, or claim that may be made by its manufacturer, is not guaranteed or endorsed by the publisher.
